# 

**Published:** 1887-07-02

**Authors:** 


### The Hospital.

**An Institution, Family, and Parochial Journal of Hospitals, Asylums, and all Agencies for the Care of the Sick, Criticism and News.**

SATURDAY, JULY 2ND, 1887.

### Questions and Answers.

All questions should be addressed to Mr. Howard J. Collins, Secretary, The Hospitals Association, Norfolk House, Norfolk Street, Strand, W.C.

T. D. W. writes: "We have a difficulty in our hospital at present about the number of resident medical officers which we should have. We have 190 beds, of which there are never more than 180 occupied, by about 100 surgical and 80 medical cases. Might I ask what you think?"

*** The number of resident medical officers varies in different hospitals, being greater where there is a medical school and the consequent necessity for clinical teaching. In all cases there should not be less than one resident officer for each fifty in-patients. At medical schools that number might be doubled with advantage.—Ed. T. H.

G. K. writes:—"Having read with much interest the accounts of the medical museums at Guy's, I should be glad to know whether the general public are admitted, or if the museums are only open to medical men. I should feel much obliged if you would kindly answer this question in The Hospital."

*** We received a similar inquiry some weeks ago, and replied to it in the number of this Journal, published on April 23rd. These museums are not, as a rule, open to the general public, but it is improbable that a special application to the Dean of the Medical College would be refused.

A correspondent writes:—"Can you tell me of any institution or hospital where men are received for training as nurses, also where lessons in massage, etc., could be obtained?"

*** Classes are held at the West-end Hospital for Diseases of the Nervous System, Paralysis, and Epilepsy, 73, Welbeck Street, London, W., on Tuesday, Wednesday, and Thursday mornings at 10 a.m. for male students. The fee for the full course (three months) of instruction in massage and electricity (including lectures by Dr. Tibbits on galvanism, and Dr. Stretch Dowse on massage, and practical massage in the wards of the hospital) is five guineas. Further particulars can be obtained from the lady superintendent.

E. Morgan, of Jersey, asks:—"Can you give me the names of any institutions where a young lady could be placed who is suffering from scrofula, so that she might be under special medical treatment?"

*** The Royal Sea-Bathing Infirmary, Margate, is the only institution in England exclusively devoted to the treatment of the scrofulous necessitous poor. The Scarborough Sea-Bathing Infirmary has, however, ten beds set apart for the treatment of such cases. It is necessary for both institutions to obtain a Governor's recommendation, and to pay the sum of £2 8s. for eight weeks' treatment (*i.e.*, six shillings per week), and to be provided with change of linen, towels, comb, and brush, etc. Out-patients are also treated at the Margate Infirmary, and are charged one shilling per week for baths, treatment, etc.

Miss L. A. Smith will be informed when the article is to appear if she will send stamped addressed envelope to the Editor.

A "District Nurse's" poetry is not without merit, but it is more suitable for a religious journal than for The Hospital. Perhaps "District Nurse" will try again; and if she does, we would advise her to deal with subjects that lie within the range of her daily experience.

### NOTICE TO CORRESPONDENTS.

All correspondence must be written on one side of the paper only.
We cannot undertake to return MSS. not used.
**Communications**, whether intended for insertion or for private information, must be authenticated by the names and addresses of the writers, not necessarily for publication.
**Local papers** containing reports or news paragraphs **should be marked.**
It is especially requested that early intelligence of **local events of interest**, or which it may be thought desirable to bring under notice, may be sent direct to the Editor.
Communications respecting editorial matters should be addressed to the Editor, Norfolk House, Norfolk Street, Strand, London, W.C.

### NOTICE TO ADVERTISERS.

*To insure prompt insertion, all Advertisements for* THE HOSPITAL *must be sent in direct to the Advertising Agent, Mr. A. P. Watt*, 2, *Paternoster Square, E.C.*

### THE LITERARY PRIZE.

A PRIZE of **Two Guineas** will be given every month for the best story or incident based upon hospital, or asylum, or student life, or any incident connected with the professions of medicine or nursing.

### THE CHILDREN'S CORNER.

**Fourth Quarterly Prize Competition.**
Begins 2nd July, 1887 : ends 24th Sept., 1887.

PRIZES.

FOR MOST CORRECT ANSWERS TO PUZZLES.

| | | | |
|---|---|---|---|
| 1st .. | .. Thirty Shillings | 3rd .. | .. Ten Shillings |
| 2nd .. | .. Twenty „ | 4th .. | .. Five „ |

FOR NEATEST ANSWERS TO PUZZLES.
Five Shillings.

FOR BEST LITTLE LETTERS.
Ten Shillings.

**Puzzles—First Week.**

No. 1. ENIGMA.

My first when harvest's o'er is seen
In every farmer's barn. I ween ;
My second always may be found
In hedges, trees, or on the ground ;
My whole in season all enjoy,
I'm welcome both to girl and boy.

No. 2. ARITHMETICAL PUZZLE. Add two figures to nine, so as to make it less than ten.

No. 3 CONUNDRUM. Why is Eau de Cologne like a foreign letter ?

No. 4. GEOGRAPHICAL CHARADE My first was made to cross my second, my whole a well-known town is reckoned.

**Letters—First Week.**

Next week, tell me your favourite way of spending a "Half-holiday."

**Results of Twelfth Week—Puzzles.**

No. 47. NATURAL HISTORY ACROSTIC.

L ark.

I bis.

N ightingale.

N uthatch.

E agle.

T hrush.

No. 48. DOUBLE MESOSTICH.

J

p U n f a B l e

J U B I L E E

l i L a c

D E e

E

No. 49. FRENCH PUZZLE. The answer to this is easily seen :
Sous Mazarin la France
A souffert mille souffrances.

No. 50. GEOGRAPHICAL TRANSPOSITION. "Ne'er match;s," ransposed, forms Manchester.

*All right*—Dot, Joe, Alsie, Tim, Umslopogaas, A. C. K., Skye ; *Three right*—The Eda, Bohemian Girl, Mikado, Nordisa, Scrooge ; *Two right*—Agatha, A. G. S. McCulloch, R. Epton, Gom, Sutton.

**Letters.**

All my small correspondents agreed this week that hay-making, in spite of drawbacks, is a very delightful pastime. Some of my little friends must have slept very soundly after their hard day's work, and that charming repast in the farm-house, "where we ate up nearly everything." Poor farmer's wife! Mikado thinks the best fun in the hayfield is a ride in the empty waggon, with a climb up the stack to finish, and a grand slide down to crown all! Here are two letters from Bohemian Girl and Skye, both pleasant to read.

DEAR MR. EDITOR,—*Haymaking* is just the summer treat I look forward to most: I love the sweet fresh smell of the hay ; it is so pleasant to make a fragrant heap to lie upon, and what is more delightful than a regular hayfield tea with strawberries and cream, and bread and butter, and fun ? I think, however hard, work men and women may consider hay-making, they really must enjoy it, though the sun may be hot.
Yours truly, BOHEMIAN GIRL, aged 13.
12, Upper Tichborne Street, Leicester.

DEAR MR. EDITOR,—I do wish you had put off your order for "Haymaking" for a fortnight, for I had the prospect of a day in the hayfield by then ; but Jubilee has put everything else out of people's heads this week. The last haymaking I had was in Cumberland, in sight of Crossfell. My brother and I worked hard nearly all day. It was very nice tossing and gathering and raking. My hands were blistered for days after, but I did not mind that much. It was very pleasant. The breeze from the mountains, and the fresh smell of the newly-cut hay and clover were too pleasant to forget. Then we all went to tea at the farmer's house. We were so hungry I think we ate up everything. It is very amusing to see some people make hay. They hold the rake as if it were a pen or pencil. There is no fun like haymaking to those who can enter into the spirit of the work.—Yours faithfully, SKYE.
4, Milton Terrace, New Road, Chatham.

Skye's description of some amateur haymaking is very funny. The Eda gives me a good account of a pleasant day spent near Loch Lomond.

**Notices to Correspondents.**

BOHEMIAN GIRL.—Many thanks for your conundrum, which I hope is an original one.

SKYE.—Your puzzle seems a very good one. Many thanks for it.

THE EDA and R. EPTON.—Each light as well as the whole word must be sent with the Acrostics and Mesostich puzzles.

NORDISA.—Answers must be addressed *Puzzle Editor*.

AGATHA.—Your wish shall be attended to.

**Rules.**

Only one side of the paper should be written upon.

The address and full Christian name and surname, and the age of the competitor, must be stated in all replies. A *nom de plume* may be added for the purposes of publication.

The first prize may not be taken by the same competitor more than once in any one year. In the event of the winner of the first prize again scoring highest, the second prize will be awarded, the next competitor receiving the first.

Answers must be addressed to the "PUZZLE EDITOR," THE HOSPITAL, Norfolk House, Norfolk Street, Strand, W.C., not later than Thursday next. The "Children's Corner" Coupon (see advertisement pages) must be enclosed.

### AMUSEMENTS WITH PROFIT.

### PRIZE COMPETITIONS.

**Quarterly Prize Competition.**
Began 23rd April ; ends 16th July.

**A Prize of Three Guineas**

will be awarded to the Competitor who shall have sent in the largest number of correct or best answers. No one will be permitted to win the Three Guinea Prize twice in any one year, but we shall award annually an additional

**Prize of Five Guineas**

to the competitor who shall have sent in the largest number of correct or best answers during the twelve months.

**No. 20. Double Acrostic.**

"Of late upon historic hills with loyal light we glowed
In honour of our Queen."

1. A scene where mirth and music reign,
2. A Turk can this distinction gain.
3. The country has been this of late,
4. A mode of transit out of date.
5. An authoress well known to fame,
6. One who from Arctic regions came.

**No. 21. Loyalty.**

Give a definition of Loyalty. Credit will be given for answers evincing originality of thought and brevity in treatment.

**Answers.**

No. 17. DOUBLE ACROSTIC.

V espasia N

I ndi A

C hi P

T obacc O

O i L

R ac E

I nig O

A nacreo N

Best answers have been received from Tabs, Ellice, Dawnlover, Boss, Gretta, Letheador, May, A. M. E., Mater, Mrs. Bedingfeld.

K. M.—You have two "lights" wrong.

CANICE.—One "light" wrong.

No. 18. CHARADE.

Ear-nest = Earnest.

Correct answers have been received from Boss, Gretta, Ellice, K. M., Canice, Tabs, Dawnlover, Arale, Letheador, May, A. M. E., Hexameter, Mater, Mrs. Bedingfeld.

**Notices to Correspondents.**

ELLICE.—In reply to your inquiry, yours was one of the answers credited.

LETHEADOR.—Other correspondents gave "this light" correctly, but failed in others, so their answers could not be credited. In all puzzles some ambiguity of expression is allowed. When two solutions suggest themselves it is better to send both.

Answers must be addressed to THE PRIZE EDITOR, AMUSEMENTS WITH PROFIT, THE HOSPITAL, Norfolk House, Norfolk Street, Strand, W.C., not later than the *first* post on Friday next. The full name and address must in all cases be given, but a *nom de plume* may be added for publication. Only one side of the paper should be written on. The "Amusements with Profit" Coupon (see advertisement pages) must be enclosed with replies.

